# Parental bonding in males with adjustment disorder and hyperventilation syndrome

**DOI:** 10.1186/1471-244X-12-56

**Published:** 2012-06-06

**Authors:** For-Wey Lung, Ting-Hsuan Lee, Mei-Feng Huang

**Affiliations:** 1 Songde Branch, Taipei City Hospital, Taipei, Taiwan; 2 Department of Psychiatry, National Defense Medical Center, Taipei, Taiwan; 3 Department of Psychiatry, Kaohsiung Armed Forces General Hospital, Kaohsiung, Taiwan; 4 Calo Psychiatric Center, Pingtung County, Taiwan; 5 Department of Psychiatry, Kai-Suan Psychiatric Hospital, Kaohsiung, Taiwan; 6 Department of Neurology, Kaohsiung Medical University, Kaohsiung, Taiwan

**Keywords:** Adjustment disorder, Hyperventilation syndrome, Parental attachment, Multitrait methodology, Hierarchical structural equation model

## Abstract

**Background:**

The purpose of the study was to identify the style of parental bonding and the personality characteristics that might increase the risk of hyperventilation and adjustment disorder.

**Methods:**

A total of 917 males were recruited, 156 with adjustment disorder and hyperventilation syndrome (AD + HY), 273 with adjustment disorder without hyperventilation syndrome (AD–HY), and 488 healthy controls. All participants completed the Parental Bonding Instrument, Eysenck Personality Questionnaire, and Chinese Health Questionnaire.

**Results:**

Analysis using structural equation models identified a pathway relationship in which parental bonding affected personality characteristics, personality characteristics affected mental health condition, and mental health condition affected the development of hyperventilation or adjustment disorder. Males with AD–HY perceived less paternal care, and those with AD + HY perceived more maternal protection than those with adjustment disorder and those in the control group. Participants with AD–HY were more neurotic and less extroverted than those with AD + HY. Both groups showed poorer mental health than the controls.

**Conclusions:**

Although some patients with hyperventilation syndrome demonstrated symptoms of adjustment disorder, there were different predisposing factors between the two groups in terms of parental bonding and personality characteristics. This finding is important for the early intervention and prevention of hyperventilation and adjustment disorder.

## Background

Parental attachment and premorbid personality traits play an important role in shaping the developmental trajectory of an individual and influence their ability to adjust to stressful events [1-3]. In particular, problems with parental attachment during childhood are considered to be a predisposing factor for the onset of many psychiatric conditions, such as anxiety, depressive states, and maladjustment behaviors [4-8].

Hyperventilation syndrome refers to various somatic and psychological symptoms that appear to be a consequence of episodes of hyperventilation with no known organic basis [9]. It was identified first by DaCosta in 1871 in military personnel [10]. A study of functional somatic complaints, including hyperventilation syndrome, found that adolescents with these complaints have poor psychosocial adjustment to negative life events [11]. It has been found that this is related to overwhelming anxiety, distressful events, military life, and other situations [12]. Moreover, people with hyperventilation syndrome tend to have high levels of obsessional behavior, together with difficulty handling everyday stress [13]. The diagnostic category of hyperventilation syndrome remains unknown; however, it has been described as a type of maladjustment behavior, and it can be comorbid with emotional problems [14].

The term adjustment disorder refers to patients who have developed clinically significant emotional or behavioral symptoms; these symptoms dissipate eventually, once the stressor has been terminated [15]. It has been argued that adjustment disorder does not conform to the criteria for specific symptoms, because approximately 35% of patients with adjustment disorder have comorbidity with other Axis I and II diagnoses [16,17]. In addition, the diagnosis of adjustment disorder is difficult to determine, and it has been described as a ‘waste basket diagnosis’.

It is thought that hyperventilation syndrome might be related to adjustment disorder. In addition to stress, both hyperventilation syndrome and adjustment disorder have been found to be associated with parental bonding and personality characteristics [1,14,18]. In these earlier studies, male patients with adjustment disorder all showed signs of parental overprotection, regardless of whether they showed symptoms of hyperventilation. In addition, those with more introverted and neurotic personality characteristics were more likely to develop adjustment disorder [14,18]. Patients who report a greater degree of paternal overprotection and less parental care are also more likely to develop adjustment disorder [1]. Thus, parental bonding and personality characteristics have a major effect on hyperventilation syndrome and adjustment disorder. In particular, attachment insecurity leads to an increased risk of disease through a range of mechanisms, which include increased susceptibility to stress, increased use of external regulators of dysphoric affect, and alterations in help-seeking behavior [19,20]. The differences between patients with adjustment disorder, those with hyperventilation syndrome, and male individuals without psychiatric illnesses are unclear; nevertheless, understanding the relationship between the two conditions is important.

Hence, the purpose of the study reported herein was to identify the style of parental bonding and the personality characteristics that might increase the risk of hyperventilation syndrome and adjustment disorder. Patients with hyperventilation syndrome, those with adjustment disorder, and male controls were compared to determine the differences between the groups, to provide information for the development of early intervention and prevention programs.

## Methods

### Participants

Ethical approval was obtained from the institutional review board at Kaohsiung Armed Forces General Hospital in southern Taiwan. The participants gave their consent after they were informed of the details of the study. The study population included 273 male military conscripts who had been diagnosed with adjustment disorder by a psychiatrist at a teaching hospital in southern Taiwan. An additional 156 male conscripts with adjustment disorder who visited the emergency room at the same hospital because of an episode of acute hyperventilation were also recruited. First, all participants who attended the emergency room underwent several clinical assessments, which included taking a history, a physical examination, noninvasive blood pressure measurement, 12-lead ECG, continuous ECG monitoring, pulse oximetry, standard blood tests, and chest-X ray. These assessments were performed by physicians in the emergency department. Second, arterial blood was withdrawn to measure the partial pressure of carbon dioxide (PCO_2_) (see Table 1). The diagnosis of acute hyperventilation was made by two internal medicine specialists on clinical grounds, on the basis of the patient history, clinical observation, physical examination, and biochemical tests. Subsequently, a psychiatrist was informed so that they could take over the care of the patient after organic medical diseases had been ruled out. Finally, all patients with hyperventilation syndrome were interviewed by a senior psychiatrist, and those who were also diagnosed with adjustment disorder were assigned to the AD + HY group.

**Table 1:** **The pH, O**_**2**_**, and CO**_**2**_**values in participants with hyperventilation syndrome**

	**M**	**SD**	**Min**	**Max**
pH	7.49	0.02	7.36	7.65
O_2_ value	57.31	7.93	21.9	130.1
CO_2_ value	29.76	2.02	13.7	51.6

All participants in the adjustment disorder with hyperventilation (AD + HY) group and in the adjustment disorder without hyperventilation (AD–HY) group were admitted to a teaching hospital for at least one week to verify the diagnosis. The patients were interviewed by a senior psychiatrist with reference to the *Diagnostic and Statistical Manual of Mental Disorders, Fourth Edition* (DSM-IV) [15]. All of the participants had presented without any pre-existing mental disorders. No participant who had any clinical evidence of physical illness, panic disorder, or other psychiatric disorders included in the *International Classification of Diseases, 10th Edition* (ICD-10) [21] was referred. A one-year cohort study was also used to verify the stability of the diagnosis of adjustment disorder.

A control group composed of 488 male conscripts who were at the same military base as the case group and had no experience of hyperventilation syndrome or any other physical or psychiatric illness was also recruited.

### Materials

A self-report questionnaire, which included demographic information, the Parental Bonding Instrument (PBI), the Eysenck Personality Questionnaire (EPQ), and the Chinese Health Questionnaire (CHQ), was used. The demographic data collected included the patient’s age, marital status, and education.

#### Parental bonding instrument (PBI)

Parker, Tupling, and Brown developed the PBI in 1979 [22]. The PBI was designed as a refined self-report measure of fundamental dimensions of care and protection for each parent. The Chinese version of the PBI was modified by Shu, Lo, and Lung [23]. The questionnaire instructs respondents to rate “how true” they judge each of the 25 items as a description of their mother’s and (separately) father’s behaviors toward them during the first 16 years of life. The rating options are “very much like”, “moderately like”, “moderately unlike” and “very much unlike”, which generate scores of 3, 2, 1, and 0, respectively. The Cronbach alpha value for the PBI is 0.65–0.73, and test–retest reliability is 0.66–0.88 [23].

#### Eysenck personality questionnaire (EPQ)

Eysenck and Eysenck developed the EPQ [24]. It consists of four subscales: psychoticism, extroversion, neuroticism, and lying. The Chinese version of the EPQ was modified by Lu [25] and includes 25 items, which comprise two subscales: extroversion (14 items) and neuroticism (11 items). A Cronbach alpha value of 0.90 and good validity were demonstrated by Lu [25].

#### Chinese health questionnaire (CHQ)

Cheng and Williams [26] designed the CHQ. It was derived from a Chinese translation of the General Health Questionnaire [27,28], with culturally relevant items added to a primary item pool. This item pool was treated with discriminant function analysis to select a subset of 12 items. A simple scoring method of 0-0-1-1, with rating options of “not at all”, “same as usual”, “more than usual”, and “a lot more than usual”, is applied to the CHQ. The optimum cutoff point (the best compromise between high sensitivity and a low false-positive rate) for the CHQ, as determined using the receiver operating characteristic (ROC) curve, is 3/4 [29,30]. The internal consistency of the CHQ is indicated by an alpha coefficient of 0.79, as demonstrated by Cheng et al. [29].

### Statistical analysis

The data were analyzed using the software package SPSS 17.0 for Windows (SPSS, Chicago, IL), and the structural equation model (SEM) was analyzed using the AMOS 7.0 statistical software package (SPSS, Chicago, IL). All variables were analyzed using one-way analysis of variance (ANOVA) to compare the differences among the three groups. In addition, Bonferroni post hoc multiple comparisons were used to compare each pair group in terms of any statistically significant variables. The SEM techniques made use of all the information that was provided by the regression techniques in a path analysis. If the SEM resulted in a p value greater than 0.05, an adjusted goodness-of-fit index (AGFI) greater than 0.9, and a root mean square error of approximation (RMSEA) less than 0.08, this indicated that the null model corresponded to the real structure.

## Results

The demographic distribution of the three groups – those with adjustment disorder and hyperventilation (AD + HY), those with adjustment disorder alone (AD–HY), and controls – was as follows. All participants were male; the mean age of the AD + HY group was 21.12 (SD = 1.98) years, for the AD–HY group it was 21.86 (SD = 2.36) years, and for the controls 22.61 (SD = 2.18) years. In terms of the average years of education received by the participants, all had graduated from high school; the AD + HY group had received a mean of 12.92 (SD = 2.29) years of education, the AD–HY group a mean of 13.56 (SD = 2.87) years, and the controls 13.94 (SD = .61) years. There were statistically significant differences with respect to age (F = 29.91, *p* < 0.001) and level of education (F = 8.93, *p* < 0.001) among the three groups; in subsequent analyses, these factors were controlled for.

### One-way ANOVA

The comparisons of parental bonding, personality characteristics, and mental health condition among the three groups are shown in Table 2. The results of ANOVA showed statistically significant differences among the three groups with respect to all dimensions measured. The differences in paternal and maternal care among the three groups, as assessed with the PBI, were statistically significant (F_paternal care_ = 73.02, *p* < 0.001; F_maternal care_ = 34.29, *p* < 0.001). For paternal care, in the AD + HY group the mean score was 19.07 (SD = 6.88), and in the AD–HY group it was 16.59 (SD = 7.48), compared with 22.86 (SD = 6.78) in the controls. For maternal care, in the AD + HY group the mean score was 22.75 (SD = 6.04), and in the AD–HY group it was 21.34 (SD = 7.63), compared with 25.42 (SD = 6.39) in the controls. The differences in paternal and maternal protection among the three groups were also statistically significant (F_paternal protection_ = 66.49, *p* < 0.001; F_maternal protection_ = 48.76, *p* < 0.001). For paternal protection in the AD + HY group the mean score was 14.47 (SD = 5.54), and in the AD–HY group it was 15.50 (SD = 6.20), compared with 10.68 (SD = 5.81) in the controls. For maternal protection in the AD + HY group the mean score was 14.72 (SD = 6.54), and in the AD–HY group it was 15.29 (SD = 6.89), compared with 10.92 (SD = 6.06) in the controls. Using Bonferroni post hoc tests, between-group comparisons showed that there were no significant differences between the AD + HY and AD–HY groups in terms of the parental bonding dimensions of maternal care, maternal protection, and paternal protection (MD = 1.41, *p* = 0.110; MD = −0.56, *p* = 1.000; MD = −1.03, *p* = 0.246). The results are shown in Table 2.

**Table 2:** Parental attachment, personality characteristics, and mental health status of patients with AD + HY, with AD–HY, and controls (N = 917)

**Factors**	**AD + HY**	**AD–HY**	**Control**	**ANOVA**	**Bonferroni post hoc test**
	**(n = 156)**	**(n = 273)**	**(n = 488)**		
	**Mean (SD)**	**Mean (SD)**	**Mean (SD)**		
Personality characteristics					
Extroversion	6.08 (4.23)	4.47 (3.94)	9.31 (3.50)	F = 155.53	Hyperventilation < Control; MD = −0.32, *p* < 0.001
				*p* < .001	Hyperventilation > Adjustment; MD = 1.61, *p* < 0.001
					Adjustment < Control; MD = −4.85, *p* < 0.001
Neuroticism	7.86 (3.08)	9.26 (2.34)	3.00 (3.04)	F = 473.74	Hyperventilation > Control; MD = 4.86, *p* < 0.001
				*p* < .001	Hyperventilation < Adjustment; MD = −1.39, *p* < 0.001
					Adjustment > Control; MD = 6.26, *p* < 0.001
Parental bonding					
Maternal care	22.75 (6.04)	21.34 (7.63)	25.42 (6.39)	F = 34.29	Hyperventilation < Control; MD = −2.67, *p* < 0.001
				*p* < .001	Hyperventilation > Adjustment; MD = 1.41, *p* = 0.110
					Adjustment < Control; MD = −4.08, *p* < 0.001
Maternal protection	14.72 (6.54)	15.29 (6.89)	10.92 (6.06)	F = 48.76	Hyperventilation > Control; MD = 3.81, *p* < 0.001
				*p* < .001	Hyperventilation < Adjustment; MD = −0.56, *p* = 1.00
					Adjustment > Control; MD = 4.37, *p* < 0.001
Paternal care	19.07 (6.88)	16.59 (7.48)	22.86 (6.78)	F = 73.02	Hyperventilation < Control; MD = −3.79, *p* < 0.001
				*p* < .001	Hyperventilation > Adjustment; MD = 2.48, *p* = 0.001
					Adjustment < Control; MD = −6.27, *p* < 0.001
Paternal protection	14.47 (5.54)	15.50 (6.20)	10.68 (5.81)	F = 66.49	Hyperventilation > Control; MD = 3.79, *p* < 0.001
				*p* < .001	Hyperventilation < Adjustment; MD = −1.03, *p* = 2.47
					Adjustment > Control; MD = 4.82, *p* < 0.001
Mental health	6.62 (3.51)	7.45 (3.49)	1.00 (1.88)	F = 574.44	Hyperventilation > Control; MD = 5.63, *p* < 0.001
				*p* < .001	Hyperventilation < Adjustment; MD = −0.83, *p* = 0.008
					Adjustment > Control; MD = 6.46, *p* < 0.001

AD + HY: adjustment disorder with hyperventilation syndrome; AD–HY: adjustment disorder without hyperventilation syndrome; MD: mean difference.

The mean score for extraversion in the AD + HY group, as assessed with the EPQ, was 6.08 (SD = 4.23), and in the AD–HY group it was 4.47 (SD = 3.94), compared with 9.31 (SD = 3.50) in the controls. The mean score for neuroticism in the AD + HY group was 7.86 (SD = 3.08), and in the AD–HY group it was 9.26 (SD = 2.34), compared with 3.00 (SD = 3.04) in the controls. The differences in extraversion and neuroticism among three groups were statistically significant (F_extraversion_ = 155.53, *p* < 0.001; F_neuroticism_ = 473.74, *p* < 0.001).

Furthermore, the mean score for mental health in the AD + HY group, as assessed with the CHQ, was 6.62 (SD = 3.51), and in the AD–HY group it was 7.45 (SD = 3.49), compared with 1.00 (SD = 1.88) in the controls. The difference in mental health among the three groups was statistically significant (F = 574.44, *p* < 0.001). Using Bonferroni post hoc tests, between-group comparisons showed that there were no significant differences between the AD + HY and AD–HY groups (MD = −0.83, *p* = 0.008).

### Structural equation modeling

**Figure 1:**
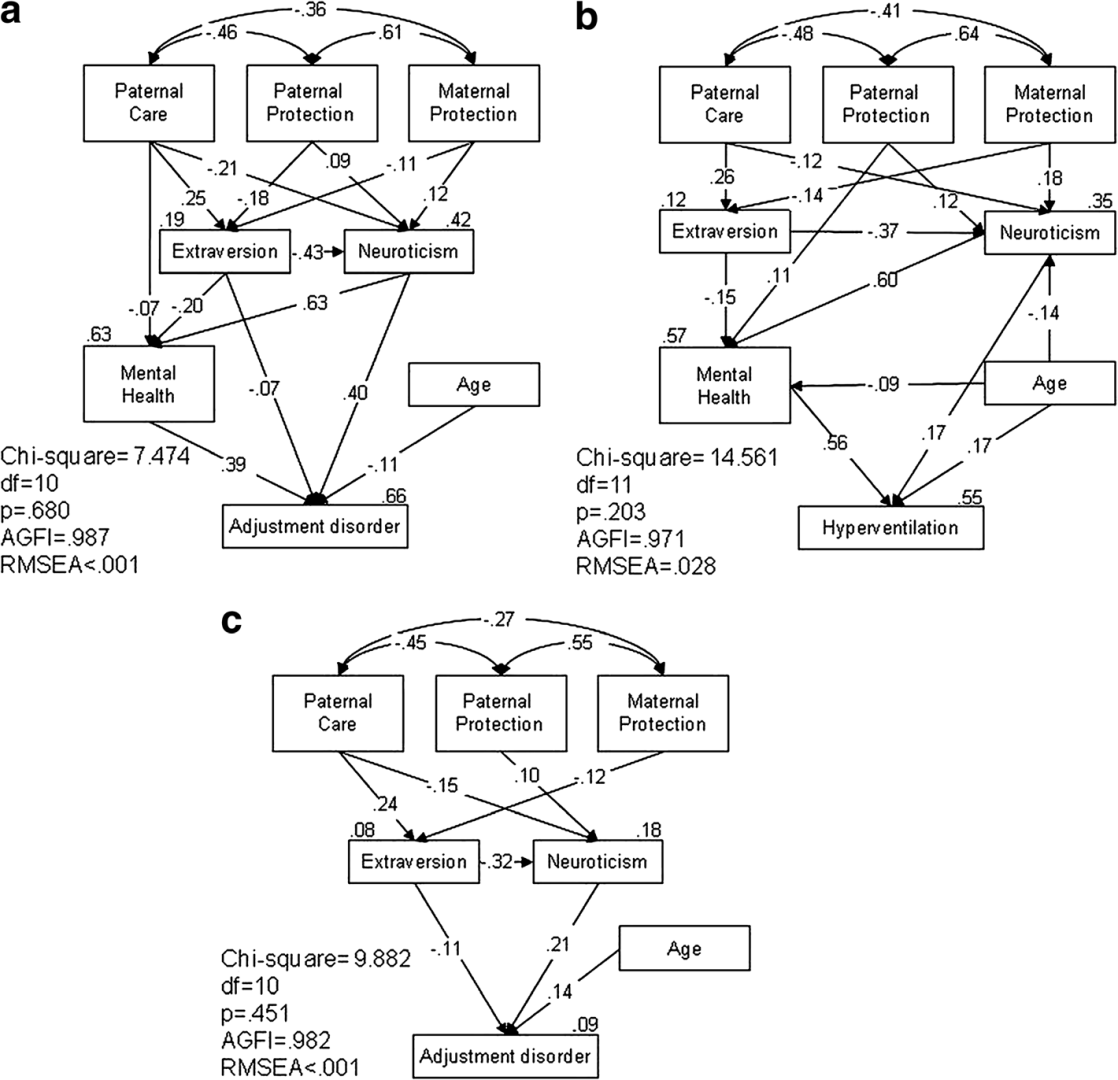
**The parsimonious structural equation model (SEM) comparison between a) the AD–HY group and controls, b) the AD + HY group and controls, and c) the AD–HY and AD + HY groups with respect to parental attachment, personality characteristics, and mental health condition.** AGFI: adjusted goodness-of-fit indices; RMSEA: root mean square error of approximation; AD + HY: adjustment disorder with hyperventilation syndrome; AD–HY: adjustment disorder without hyperventilation syndrome. a) group dummy variable of 1: AD–HY, 0: control. b) group dummy variable of 1: AD + HY, 0: control. c) group dummy variable of 1: AD + HY, 2: AD–HY.

SEM was used to investigate differences in the hierarchical pathways between parental bonding, personality characteristics, and mental health condition among the three groups. The analysis was carried out in three paired group models: AD–HY and controls in the first model (Figure 1a), AD + HY and controls in the second model (Figure 1b), and AD–HY and AD + HY in the third model (Figure 1c). Parsimonious models were developed, which meant that only statistically significant pathways were shown. All models showed a pathway relationship in which parental bonding affected personality characteristics, personality characteristics affected mental health condition, and mental health condition affected whether subjects developed adjustment disorder or hyperventilation, as shown in Figure 1.

The first parsimonious model, which compared AD–HY and controls, resulted in a good fit, with a *p* value of 0.680 (i.e., greater than 0.05), an AGFI of 0.987 (i.e., greater than 0.9), and an RMSEA of less than 0.001 (i.e., less than 0.5), as shown in Figure 1a. When fathers showed less care, their sons were less extroverted, more neurotic, and had poorer mental health (β = 0.25, *p* < 0.001; β = −0.21, *p* < 0.001; β = −0.07, *p* = 0.016, respectively). When parents were overprotective, their sons were less extroverted and more neurotic (father: β = −0.18, *p* < 0.001; β = 0.09, *p* = 0.039; mother: β = −0.11, *p* = 0.035; β = 0.12, *p* = 0.006, respectively). In addition, participants who were less extroverted or more neurotic had poorer mental health and were more likely to develop adjustment disorder (extroversion: β = −0.20, *p* < 0.001; β = −0.07, *p* = 0.023; neuroticism: β = 0.63, *p* < 0.001; β = 0.40, *p* < 0.001, respectively). Finally, participants with poorer mental health or those who were younger were more likely to develop adjustment disorder (β = 0.39, *p* < 0.001; β = −0.11, *p* < 0.001, respectively). Extroversion, neuroticism, mental health condition, and adjustment disorder accounted for 19%, 42%, 63%, and 66% of the variance, respectively.

The second model compared participants with AD + HY and the controls. The model resulted in a good fit, with a *p* value of 0.203, an AGFI of 0.971, and an RMSEA of 0.028, as shown in Figure 1b. Those who had received less paternal care and maternal overprotection were less extroverted and more neurotic (father: β = 0.26, *p* < 0.001; β = −0.12, *p* = 0.013; mother: β = −0.14, *p* = 0.006; β = 0.18, *p* = 0.001, respectively). In the case of fathers who were overprotective, their sons were more neurotic and had poorer mental health (β = 0.12, *p* = 0.029; β = 0.11, *p* = 0.002, respectively). In addition, participants who were less extroverted had poorer mental health (β = −0.15, *p* < 0.001), and those who were more neurotic had poorer mental health and a greater likelihood of developing adjustment disorder and hyperventilation (β = 0.60, *p* < 0.001; β = 0.17, *p* < 0.001, respectively). Those with poorer mental health were also at a higher risk of developing adjustment disorder and hyperventilation (β = 0.56, *p* < 0.001). Extroversion, neuroticism, mental health condition, and hyperventilation accounted for 12%, 35%, 57%, and 55% of the variance, respectively.

The last model compared participants with AD + HY and those with AD–HY. The model resulted in a *p* value of 0.451, an AGFI of 0.982, and an RMSEA of less than 0.001, which indicated a good fit, as shown in Figure 1c. The dummy variable for group was 1 for those with AD + HY and 2 for those with AD–HY. Fathers who showed less care had sons who were less extroverted and more neurotic (β = 0.24, *p* < 0.001; β = −0.15, *p* = 0.003, respectively). When fathers were overprotective, their sons were more neurotic (β = 0.10, *p* = 0.043), and when mothers were overprotective, their sons were less extroverted (β = −0.12, *p* = 0.014). Compared with those with AD + HY, those with AD–HY were less extroverted and more neurotic (β = −0.11, *p* = 0.024; β = 0.21, *p* < 0.001, respectively). Extroversion, neuroticism, and adjustment disorder accounted for 8%, 18%, and 9% of the variance, respectively.

## Discussion

Use of one-way ANOVA showed significant differences in all dimensions among the three groups; however, mediating and moderating factors, and any causal effect, have been neglected. The paired group comparison SEMs for the AD + HY, AD–HY, and control groups all revealed a pathway relationship in which parental bonding affected personality characteristics, personality characteristics affected mental health condition, and mental health condition affected the development of hyperventilation or adjustment disorder. More specifically, with respect to parental bonding, males with AD–HY perceived less care from their fathers than those with AD + HY and the controls, and males with AD + HY perceived more maternal protection than those with AD–HY and the controls. Although participants with AD–HY and those with AD + HY were both more neurotic and less extroverted than the controls, those with AD–HY were even more neurotic and less extroverted than those with AD + HY. The two groups with adjustment disorder did not differ in their mental health condition; both showed worse mental health than the controls.

The pathway relationship in which parental bonding affected personality characteristics, which in turn affected mental health condition and the development of hyperventilation syndrome or adjustment disorder, is consistent with previous studies on hyperventilation and adjustment disorders [1,14,18]. Parental emotional neglect is often related to psychiatric disorder [31]. In addition, the present study found that maternal overprotection increased the risk of hyperventilation syndrome, and less paternal care increased the risk of adjustment disorder. Previous studies have also found that hyperventilation is associated with maternal attachment and premorbid personality [14]; more specifically, maternal attachment has been found to play a unique role in male adjustment disorder with hyperventilation syndrome [14]. This can be understood easily, because maternal attachment is related to the development of problems with self-esteem and behavior, particularly maladjustment behavior [1-3,14], and might be linked indirectly to clinical diagnoses of mental health problems [32].

Attachment style is an important factor in understanding the particular ways in which individuals feel and react when stressed by illness, and is important when assessing the utilization of healthcare [33,34]. With regard to the finding that less paternal care increased the risk of adjustment disorder, previous studies have also found that negative parental attachment is related negatively to the individual’s functional morbidity, such as the inability to cope with daily stress, and problems with adjustment and social skills [35,36]. The impact of high parental control and low care tends to increase mental health problems and distress, and such individuals might experience difficulties in interacting with the environment [36-40]. Therefore, dysfunctional parenting is one of the main influences on individual personality traits and the ability to adjust [1-3]. A previous study found that adolescents who had poor communication with their fathers showed more aggressive behavior toward authority in school and had more difficulty in adjusting [41]. When these males enter military service, they are within a predominantly male environment, including their superiors. The experience of poor paternal care might be projected onto these superiors, thus augmenting the adjustment problem.

Interestingly, although high neuroticism and low extroversion were found to increase the risk of adjustment disorder or hyperventilation syndrome, individuals with AD–HY showed even greater neuroticism and less extroversion than those with AD + HY. Extroversion had only an indirect effect on individuals with AD + HY when compared with the controls. Several studies have demonstrated that neuroticism and introversion might increase the risk of mental disorders such as anxiety, depression, and maladaptive behaviors [1-3,42,43]. The present study has highlighted the difference in severity between hyperventilation syndrome and adjustment disorder.

Few studies have investigated the differences between adjustment disorder and hyperventilation syndrome; however, criteria to assist in the differential diagnosis are required. Our study compared individuals with AD–HY, those with AD + HY, and controls, and identified differences among these three groups with respect to parental bonding, personality characteristics, and mental health. Self-report measures were used for the study, which might have resulted in problems of recall bias in terms of the rating of parental attachment, and this could have influenced the results. However, studies have demonstrated that the PBI has acceptable reliability and validity [22,23]. Furthermore, participant reports of parental behavior have shown a significant association with independent reports [44,45], and twin studies have also shown a high correlation in the rating of parents [45,46]. It is thought that attachment insecurity is a determinant of physical health throughout the lifespan [19,20]. In the present study, although the PBI was used to measure parental attachment, it does not assess attachment style specifically and this might be a limitation in the current study. Hence, a more in-depth assessment of attachment, using specific self-report questionnaires or the Adult Attachment Interview, should be considered for future studies.

## Conclusions

The study showed that, although some patients with hyperventilation syndrome demonstrated symptoms of adjustment disorder, different predisposing factors were found between the two groups of patients with respect to parental bonding and personality characteristics. Patients with AD–HY perceived a greater degree of paternal care, and those with AD + HY perceived a greater degree of maternal overprotection. Those with AD–HY also showed a higher degree of neuroticism and less extroversion than those with AD + HY, although both had poorer mental health than the controls. In addition, the comparison of the AD + HY group with the controls using SEM yielded high variances of 66% and 55%. With adjustment for age, sex, and level of education, this finding is potentially important for the development of early intervention and prevention programs for hyperventilation syndrome and adjustment disorder. Further studies are required to determine whether there is a gender difference in the etiology of hyperventilation syndrome and adjustment disorder, and thus whether this model can be generalized to female patients with hyperventilation syndrome or adjustment disorder.

## Competing interests

All authors have no conflict of interest to declare.

## Authors’ contributions

FWL contributed to the conceptual design of the study and modified the manuscript. THL participated in the conceptual design, collected the data, data analysis of the study, and drafted the manuscript. MFH interpreted the data. All authors read and approved the final manuscript.

## Pre-publication history

The pre-publication history for this paper can be accessed here:

http://www.biomedcentral.com/1471-244X/12/56/prepub
